# Idiopathic Intracranial Hypertension in a Morbidly Obese Young Female Managed With Bariatric Surgery

**DOI:** 10.7759/cureus.32868

**Published:** 2022-12-23

**Authors:** Alhanouf A Alkhuwaylidi, Alanoud A Alkhurayji, Bassam Alhassan, Bandar Ali

**Affiliations:** 1 College of Medicine, Princess Nourah bint Abdulrahman University, Riyadh, SAU; 2 General Surgery, Prince Sultan Military Medical City, Riyadh, SAU

**Keywords:** laparoscopic sleeve gastrectomy, visual impairment, effect of bariatric surgery, idiopathic intracranial hypertension (iih), obesity

## Abstract

Idiopathic intracranial hypertension (IIH) is a disorder of unknown etiology characterized by raised intracranial pressure (ICP) and papilledema, also known as pseudotumor cerebri. Morbid obesity mainly affects women of childbearing age, which makes it a consistent risk factor for the development of IIH in these individuals. Consequently, the higher the body mass index (BMI) the higher the risk of IIH. We report a case of a 30- year-old female with symptomatic idiopathic IIH and malfunctioning ventriculoperitoneal (VP) shunt, associated with obesity, who underwent a bariatric sleeve gastrectomy, which led to weight loss and a significant improvement in her IIH. Our objective is to better understand the efficacy of bariatric surgery as a treatment for IIH.

## Introduction

Idiopathic intracranial hypertension (IIH) is a debilitating disease and a significant cause of blindness among patients. Its incidence has increased steadily over the past few years [1]. It is usually seen in obese women, and patients generally complain of severe headaches, pulsatile tinnitus, nausea, vomiting, photophobia, radicular pain, and progressive loss of vision [1]. Furthermore, obesity is a major risk factor for IIH, and usually, the main symptom of IIH is a headache (84%) manifesting as a daily bilateral, frontal, retro-ocular headache, as well as visual loss presenting as a major morbidity leading to blindness; up to 68% of IIH patients suffer from transient visual disturbances [2]. Obesity is a serious public health issue, and over half a billion people are affected worldwide [3]. The World Health Organization (WHO) defines obesity as having a body mass index (BMI) greater than 30 kg/m^2^, which is a risk factor for a variety of comorbid conditions such as type two diabetes mellitus, malignancy, and high blood pressure, as well as premature death. Thus, obesity has an impact on all body systems, including the nervous system [3]. However, weight loss and consequent reduction in BMI have been shown to reduce intracranial pressure (ICP) and relieve the symptoms of IIH. Bariatric surgery for BMI reduction is associated with improvement in IIH symptoms and a decrease in ICP [4]. Furthermore, there is a lot of evidence to suggest that the use of bariatric surgery in people with IIH and high BMI as a management modality and the consequent weight reduction can lead to improvement in other obesity-related comorbidities as well [4]. Hence, we believe bariatric surgery is an effective method for managing obesity and resolving related comorbidities such as IIH in the long term [5].

## Case presentation

**Figure 1:**
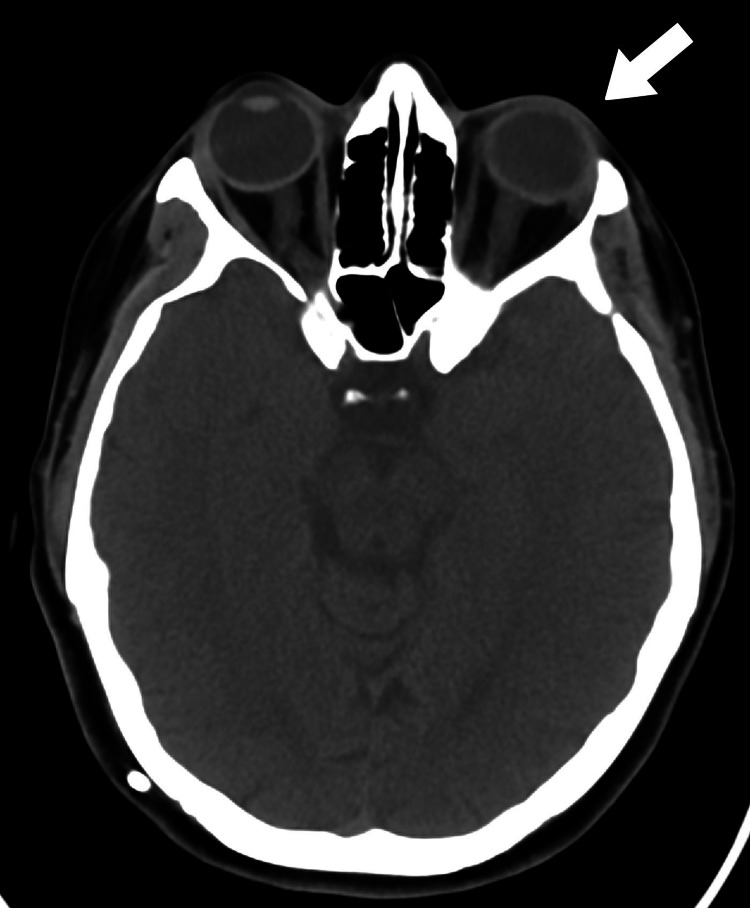
CT axial view The image shows the bilateral enlargement of the optic nerve and perioptic complex with flatting of the optic disc more on the left side with an increase in perioptic cerebrospinal fluid spaces (white arrow) CT: computed tomography

The patient was a 30-year-old female who was referred to the bariatric clinic from neurology; she was a known case of IIH with a malfunctioning ventriculoperitoneal (VP) shunt and significant visual compromise associated with a BMI of 44 kg/m^2^. The patient had been complaining of progressively worsening headaches since 2013, which occurred multiple times per week and were associated with nocturnal awakenings. The headache was not relieved by analgesics. Furthermore, it was associated with progressive loss of vision in the right eye and severe field defects in the left eye. Cranial CT had revealed pseudotumor cerebri with significant compression on optic nerves with thickening and perioptic edema bilaterally with no significant change in ventricular size, mass effect, midline shift, or intracranial hemorrhage (Figure 1).

Afterward, a VP shunt had been inserted with a programmable valve nine years ago. However, the symptoms had not improved after two weeks of insertion as the patient presented to the ER complaining of moderate headache and subjective fever, which led to the reprogramming of the shunt valve from level three to level two for more CSF drainage. The next follow-up after two weeks revealed mild improvement in her headache but the patient had been still symptomatic. Also, there was a stable vision loss in the right eye and a deterioration of the left eye vision as per ophthalmology. Hence, she was referred to the bariatric clinic. The patient was fully aware of her condition and pre-bariatric pathways, and she was seen by a dietitian, psychologist, and health educator as part of the pre-bariatric surgery assessment. After that, the patient was admitted for laparoscopic sleeve gastrectomy (LSG) with a BMI of 45.7 kg/m^2^ (weight: 123 kg, height: 164 cm).

**Figure 2:**
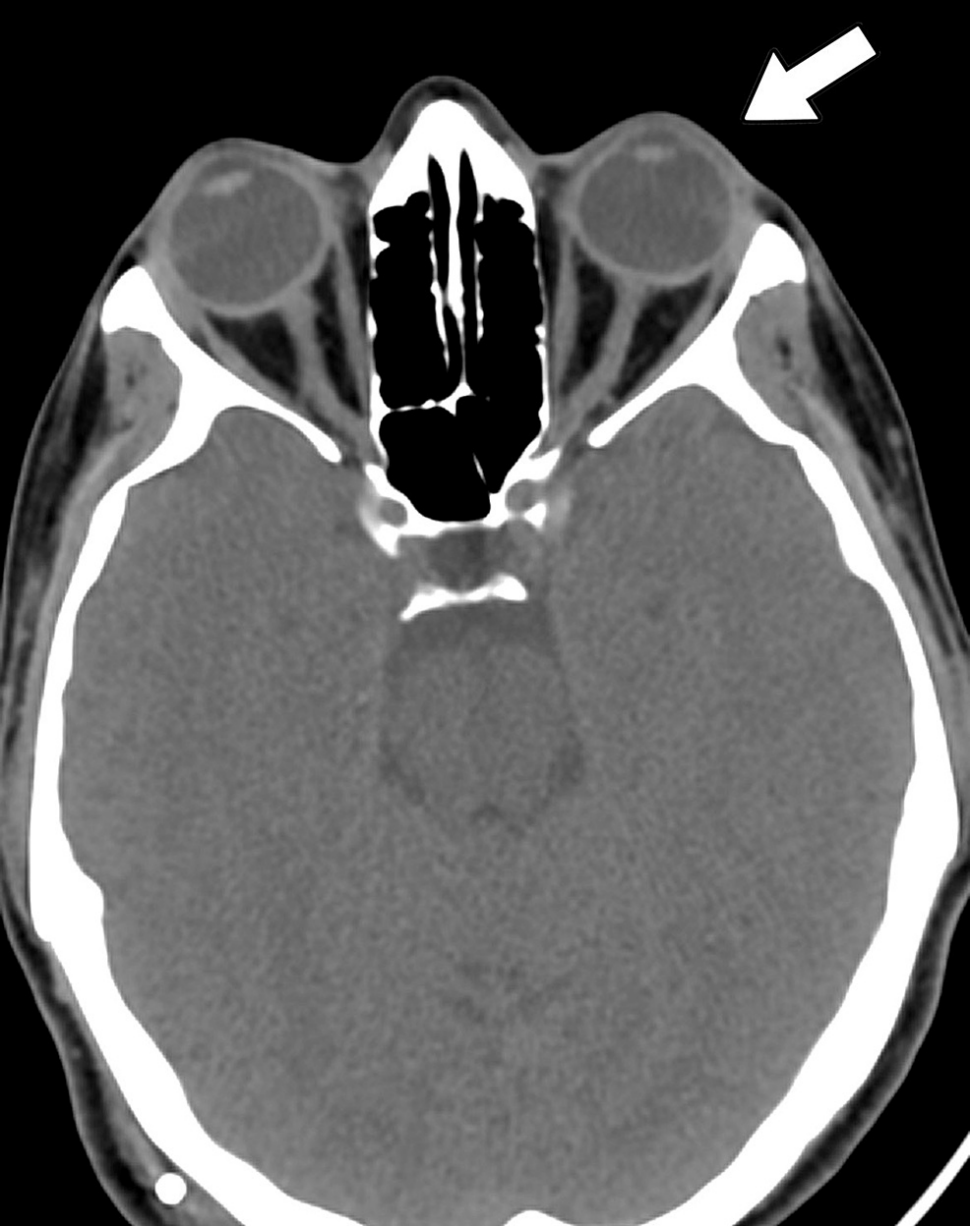
Postoperative CT axial view showing interval normalization (white arrow) CT: computed tomography

LSG was performed safely without any complications and the patient was safely discharged home on postoperative day one with postop instructions and advised to attend an outpatient follow-up. She was seen after three months of the surgery in the clinic, and showed a 28-kg weight loss, resulting in a BMI drop to 37 kg/m^2^. After the weight reduction, the headache had improved significantly, in addition to the decrease in her ICP, and her VP shunt had started to work properly. Furthermore, her symptoms of IIH resolved after the bariatric surgery except for visual impairment (Figure 2).

## Discussion

We reported a case of a morbidly obese female with IIH who did not respond to a VP shunt insertion but whose condition significantly improved after undergoing LSG, with the exception of her visual impairment. Although the underlying cause of IIH is unknown, some theories suggest that increased intra-abdominal pressure causes a cascade of increasing pressures inside the pleura, atria, and ventricles [6]. Currently, regarding the management of the condition, there is no such thing as a most effective strategic approach for IIH; however, the priorities are to maintain visual function and reduce long-term headaches [7]. Also, weight loss seems to be the only known disease-modifying method for reversing IIH [8]. Hence, bariatric surgery is a low-risk and successful therapy for obesity [9]. Moreover, it is associated with improvement in IIH, based on level II evidence [9]. Additionally, a systematic review by Manfield et al. has reported that surgical interventions have been accompanied by 100% postoperative IIH resolution versus 66.7% in the non-surgical group (95% CI: 45.6-87.8; p=0.005) [10].

The above-mentioned review discussed a study involving 65 patients with a mean pre-interventional BMI of 48.3 kg/m^2 ^who underwent bariatric surgery. The BMI decreased by 17.5 kg/m^2^ on average after bariatric surgery, which led to the complete resolution of papilledema in all reported cases (indicating relief from high ICP) and a major improvement in headache symptoms. The review described another study where 277 participants underwent non-surgical interventions for weight reduction with a pre-intervention BMI of 37.7 kg/m^2^. The results showed that BMI decreased by a weighted mean of 4.2 kg/m^2^ [10]. Even though there were variations in baseline characteristics in patients between surgical and non-surgical studies, surgical studies were found to be superior in terms of both outcomes of weight loss and clinical improvement in IIH symptomatology [10]. In addition, the mentioned study represents the largest quantifiable series to evaluate the role of weight loss on IIH in the literature so far, and its findings ensure that studies that involve the bariatric surgical approach show a larger effect on IIH outcomes compared to those involving non-surgical approaches [10]. However, these studies were not comparable, and hence their findings cannot be used to directly compare the outcomes of bariatric and non-surgical weight loss interventions [10]. Another study has presented more accurate results by comparing non-surgical and surgical methods to reduce BMI and improve symptoms of IIH; this well-conducted systematic analysis with a large sample size has convincingly demonstrated the advantage of bariatric surgical interventions compared to non-surgical interventions in managing IIH based on a significant and steady reduction in the patient's BMI following the surgical approach [1]. However, despite the fact that there is a clear correlation between IIH and obesity, IIH is not generally accepted as an indication for bariatric surgery [1]. Therefore, these patients will experience longer waiting periods for bariatric surgery, particularly in public health systems in which patients are frequently evaluated depending on their degree of obesity and comorbidity [1]. Treating IIH patients promptly with bariatric surgery could be enormously beneficial for this patient population as it could help prevent permanent blindness [1].

## Conclusions

Obesity is a condition that can lead to increased morbidity and mortality. There is an association between morbid obesity and IIH, which could lead to numerous complications such as visual impairment if not treated early. Hence, bariatric surgery has a beneficial impact in decreasing ICP, which improves the quality of life of patients with IIH associated with obesity and reduces its complications. We recommend that patients with high IIH associated with obesity be referred to a bariatric clinic for assessment to investigate if these patients might benefit from bariatric procedures.
